# Supplementary material for: Searching for phylogenetic patterns of Symbiodiniaceae community structure among Indo-Pacific Merulinidae corals
Source: PeerJ. 2019 Sep 13;7:e7669. doi: 10.7717/peerj.7669 (PMC6746223; doi:10.7717/peerj.7669)
Supplement: Figure S1 — Analysis of endosymbiont communities in Merulinidae corals based on the Bray–Curtis dissimilarity (A; stress=0.081) and Jaccard distance (B; stress=0.098) measures of symbiont types detected in at least two of three polymerase chain reactions (each ≥2 reads). Symbols are distinguished according to host species and locality. [file peerj-07-7669-s001.pdf]

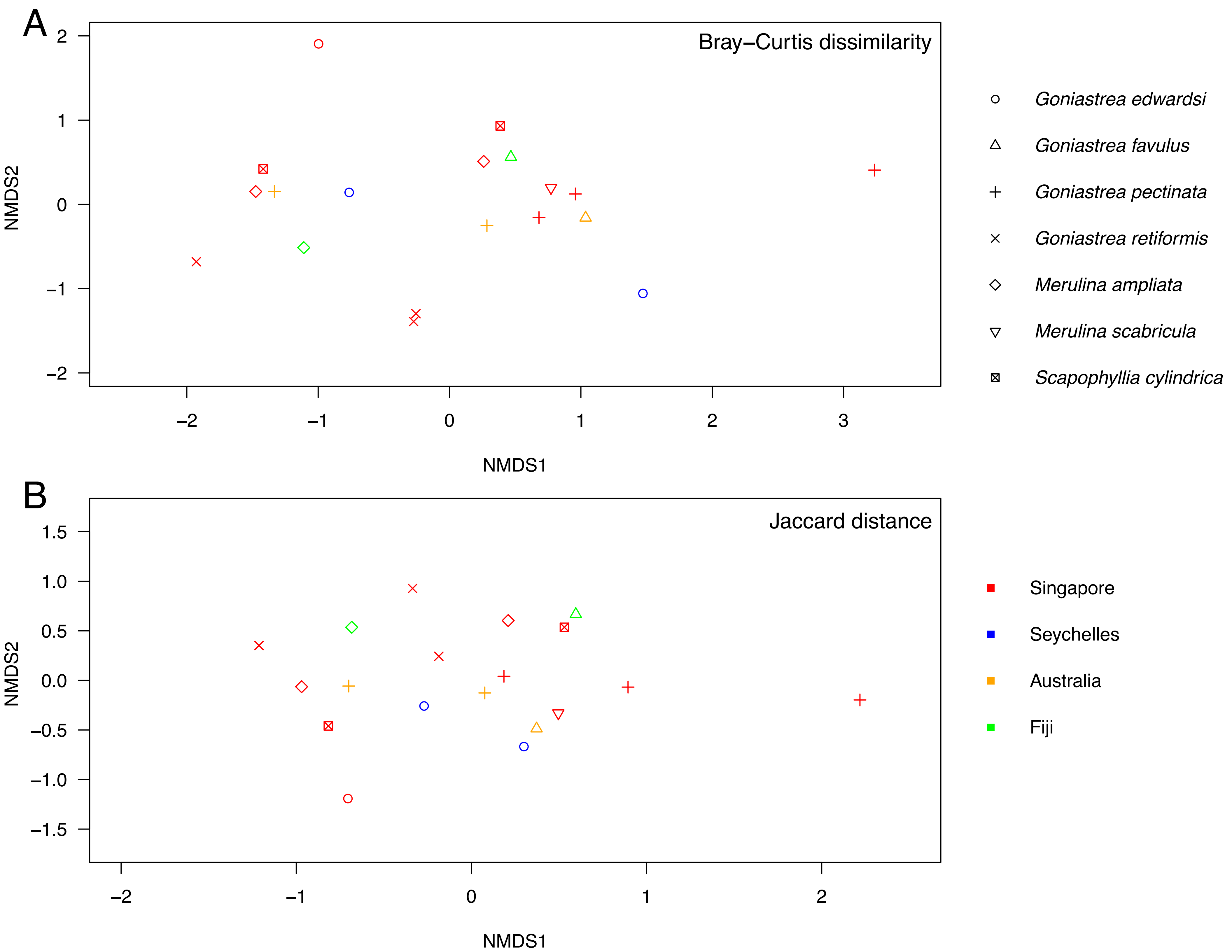

A
Bray–Curtis dissimilarity
NMDS2
NMDS1
2
1
0
−1
−2
3
Goniastrea edwardsi
Goniastrea favulus
Goniastrea pectinata
Goniastrea retiformis
Merulina ampliata
Merulina scabricula
Scapophyllia cylindrica
B
Jaccard distance
1.5
1.0
0.5
0.0
−0.5
−1.0
−1.5
Singapore
Seychelles
Australia
Fiji
