# Supplementary figures and images for: Searching for phylogenetic patterns of Symbiodiniaceae community structure among Indo-Pacific Merulinidae corals
Source: PeerJ. 2019 Sep 13;7:e7669. doi: 10.7717/peerj.7669 (PMC6746223; doi:10.7717/peerj.7669)

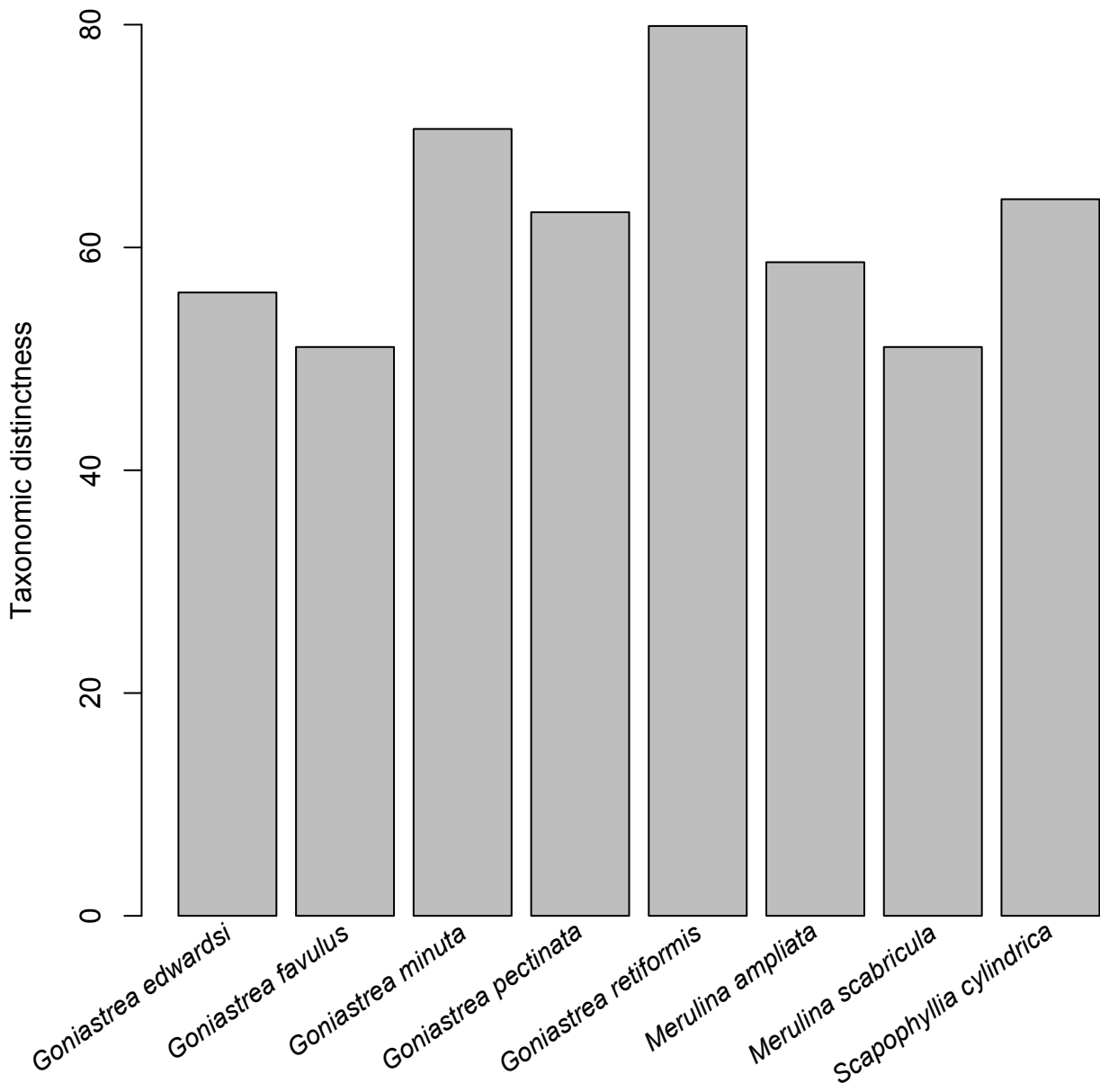

Supplement: Figure S2 — Analysis of endosymbiont communities in Merulinidae species based on the taxonomic distinctness index (Clarke & Warwick, 1998; Warwick & Clarke, 2001). [file peerj-07-7669-s002.pdf]
